# A Web-based Decision Tool to Estimate Subarachnoid Hemorrhage Risk in Emergency Department Patients

**DOI:** 10.7759/cureus.2096

**Published:** 2018-01-21

**Authors:** Haley Manella, Shyam Sivasankar, Jeffrey J Perry, Sam Pfeil, Josh Senyak, Ross Shachter, Prasanthi Govindarajan

**Affiliations:** 1 Emergency Medicine, Stanford Hospital; 2 Emergency Medicine, Dell Children's Medical Center of Central Texas; 3 Emergency Medicine, The Ottawa Hospital; 4 Management Science and Engineering, Stanford University; 5 Bioinformatics, Quicksilver Consulting

**Keywords:** sub arachnoid hemorrhage, lumbar puncture, head ct, headache

## Abstract

Subarachnoid hemorrhage (SAH) from a leaking aneurysm is a neurological emergency. SAH patients often present with headache—a common chief complaint among emergency department patients. If unrecognized, 70% of the patients with re-bleeds die and one third are left with neurological deficits. Therefore, it is critical to distinguish the signs and symptoms of SAH from benign causes of headache, perform the appropriate diagnostic tests and treat in a timely manner in order to reduce the disability and mortality associated with this condition. In patients with suspected SAH, traditional diagnostic strategies in the emergency department employ non-contrast computed tomography (CT) of the brain to detect blood in the subarachnoid space followed by lumbar puncture if there is a high clinical probability of aneurysmal bleed without any evidence of blood on CT scan. While the older generation CT scanners were less sensitive to blood detection in the subarachnoid space, recent advances in CT imaging have resulted in sensitivity approaching 100% for detection of blood in the subarachnoid space specifically within six hours of symptom onset. Therefore, the benefit of lumbar puncture is controversial when performed within the first six hours of symptom onset. Despite this, lumbar puncture is still commonly performed in the emergency department, exposing patients to unnecessary procedural risks. The objective of this research study is to develop a web-based risk calculator that estimates the risk of SAH based on time to emergency department presentation after symptom onset, physical findings and imaging characteristics with the goal of reducing unnecessary lumbar punctures in the emergency department. In this technical report, we describe the prototype calculator, the mathematical basis of the model and provide a link to the web-based prototype. In the future, we will refine the prototype, make it user-friendly to physicians, staff and patients and study its benefits in the emergency department.

## Introduction

Aneurysmal subarachnoid hemorrhage (SAH) most commonly occurs due to a ruptured cerebral aneurysm. One of the presenting symptoms of SAH is acute headache. Some of the other high-risk features identified in a cohort of SAH patients include rapidity of headache onset often described as a “thunder-clap” headache that peaks at headache onset or reach severity within minutes to an hour of onset. Headache may be associated with exertion, neck pain and/or vomiting [1, 2]. The 24-hour mortality is about 25% and if untreated, the 90-day mortality can be as high as 50%. Missed opportunities to diagnose SAH in a variety of settings including the emergency department, often lead to re-bleeding which results in higher mortality (up to 70%) [2]. Recent data looking at mortality trends show that incidence and case-fatality have not changed but the overall mortality has decreased over the years, in part due to better diagnostics and management of complications. In patients who survive, 10–20% become disabled with loss of functional independence [3].

Emergency physicians also encounter patients with benign causes of headache such as migraine, tension, and cluster types which account for 2% of emergency department visits. In order to differentiate benign headaches from acute headache related to SAH, physicians identify high-risk features in the history and obtain a non-contrast computed tomography (CT) to evaluate for evidence of blood in the subarachnoid space from the leaking aneurysm. If blood is not seen on the brain imaging, then a lumbar puncture (LP) is performed to evaluate for red blood cells or xanthochromia—a yellow discoloration of the spinal fluid due to the breakdown of the red blood cells [4]. Older generation CTs did not detect up to 5% of bleeds, but in recent years, improved CT technology has resulted in high sensitivity for detection of blood in the brain (100%) leading to almost no missed cases of aneurysmal bleeding in patients with a headache who underwent CT imaging within six hours of headache onset [5]. Despite these imaging advances and better diagnostic ability of CT scans, emergency physicians still perform lumbar punctures in low-risk patients. This results in one true positive finding for every 100–250 lumbar puncture performed by emergency physicians [2]. Lumbar puncture is an invasive test and so patients often fear having this test. In addition, this may result in post-dural headaches in 6–30% of patients, bleeding from local trauma to the pre-vertebral veins (particularly in those with bleeding diathesis or on anti-platelet/anticoagulant medicines), implantation of epidermal tissue, and local infection [6]. Although reducing unnecessary lumbar punctures and patient safety are priorities for the clinicians, currently, there is no national consensus data on the number of lumbar punctures to be performed to identify one case of SAH. Further, there are no tools that assist clinicians with the diagnostic strategy. Therefore, to provide an objective measure of SAH probability, LP risks to clinicians, and to facilitate risk-informed workup for SAH, we developed a web-based calculator (prototype) using data from previously published studies. The web tool will provide an estimate of risk of subarachnoid hemorrhage based on their headache onset to arrival time, presenting symptoms and imaging findings. The overarching goal of this tool is to reduce unnecessary testing and use the best available scientific evidence to achieve appropriate utilization of lumbar puncture in SAH diagnosis.

## Technical report

Description of the SHARED decision tool

The SHARED (Subarachnoid Hemorrhage Risk in Emergency Department) decision tool was developed by the principal investigator and the faculty at the Department of Management Science and Engineering at Stanford University. Briefly, this tool uses the test characteristics data (sensitivity, specificity) of the physical findings and CT to calculate the probability of SAH after the initial imaging tests. The calculator also estimates the risk of the procedure in patients with contraindications to the procedure. All of the probabilities and risks are integrated and presented as a visual display with recommendations on whether to proceed with a lumbar puncture as a diagnostic test.

We used a standard Microsoft Excel program (Microsoft Inc., Redmond, WA) [7] to build the decision tool. Data for the sensitivity, specificity of the tests were derived from recent meta-analysis and observational studies that provided the sensitivity, specificity of CT scan with and without the contrast and laboratory characteristics of the spinal fluid obtained in patients presenting with acute headache to emergency departments. The risks of performing the lumbar puncture in patients with a difficult body habitus or on medications (anti-platelets or anticoagulants) known to increase the bleeding risks after the procedure were included in the model.

Designing the SHARED decision tool

First, we computed the pre-test probability using a Naive Bayes model in which the Ottawa SAH rule will be used to independently change the SAH probability from the baseline prevalence of SAH in the emergency department population.

Second, for the imaging tests, we modeled the sensitivity of the CT scan as an exponential function [y = (.98) exp (-0.01*x)], because sensitivity is known to drop over time as the red blood cells disintegrate in the cerebrospinal fluid. The CT sensitivity values were obtained from recently published literature [7]. The pooled sensitivity of a non-contrast CT was 94% (95% CI: 91%–96%). When stratified by time, the sensitivity was 100% for <6 hours (95% CI: 98%–100%) and 89% for >6 hours (95% CI: 83%–93%) [2]. The sensitivities of the CT brain with contrast (CT angiogram) are known and do not drop quickly with time like the CT scan without contrast. Also, we derived the CT specificity from recent articles [5, 7]. Using these sensitivities and specificities, the SHARED decision tool computes the likelihood ratios for each test, the ratio of the probability of the observed test results given SAH to the probability of those test results given no SAH. Given the pretest probability of SAH and the product of the likelihood ratios, Bayes Theorem is used to compute the post-test probability of SAH.

In addition to the calculation above, the model also takes into account factors such as difficulty in performing a lumbar puncture due to patient body habitus and bleeding risks secondary to home medications such as anti-platelets and/or anti-coagulants. Each factor was assigned a weight to represent the difficulty with lumbar puncture. For difficult body habitus and use of anticoagulants, we assigned weights to represent the lower utility of the procedure due to side effects. For example, we assigned a weight of two for difficult body habitus (twice as difficult was our best estimate). While there is no data to assess difficulty with lumbar puncture in obese patients, data shows 69% of patients reported procedure failure in the patients with body mass index (BMI) > 30 [8]. In a patient with both risk factors, procedural difficulty is presented as the sum of these weights. While the weights do not change the post-test probability of the disease, it raises the physician’s threshold to perform the procedure particularly in low-risk patients.

SHARED visual display tool (prototype)

The prototype SAH calculator was developed as a web implementation of the decision tool described above. The user enters the number of hours since headache onset and selects answers (using radio buttons and checkboxes) to six questions. Based on these inputs, the calculator uses Javascript to calculate three probabilities: 1) the patient's pre-test probability of SAH, 2) patient's current (post-test) probability of SAH, and 3) the threshold probability at which advantages of lumbar puncture to diagnose SAH outweigh the disadvantages from patient characteristics. For added clarity, the calculator uses scalable vector graphics (SVG) to present a visual display (styled to resemble a mechanical gauge), calibrated with a logarithmic probability scale. The green display on the gauge means lumbar puncture is indicated based on post-test probability and patient risk factors, red display on the gauge means lumbar puncture is not indicated based on the post-test probability and patient risk factors, and a yellow intermediate zone in which patient preferences or physician judgment may play a role in the decision to perform the procedure.

## Discussion

Case-based examples and discussion

We use the following cases to demonstrate the recommendations of the decision tool:

Case 1

**Figure 1:**
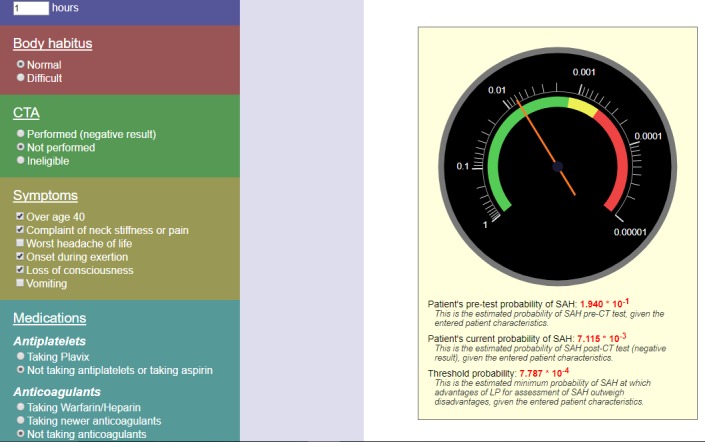
This describes the decision tool output for Case 1.

A 45-year-old male presents with headache—sudden onset with instant peaking quality and associated neck pain. He was moving heavy boxes at work when the headache began. He presented within an hour of headache onset, passed out in the triage area and had to be brought to the treatment area immediately. Upon evaluation, he was sitting upright on the gurney and was awake, alert and oriented to person, place, time and event. The patient was found to have limited neck flexion on exam. He did not have any other neurological deficits. Figure 1 displays the SHARED prototype outputs based on this case. Per the SAH Ottawa rule, this patient has multiple high-risk features including: sudden onset, instant peaking headache, onset with exertion and loss of consciousness. Based on the history and physical exam findings this patient has a high pre-test as well as post-test probability of SAH as shown on the tool. Note that the tool would recommend performing an LP if the noncontrast CT head is negative. However, if CT angiogram was performed and negative for aneurysm, the tool does not recommend lumbar puncture.

Case 2

**Figure 2:**
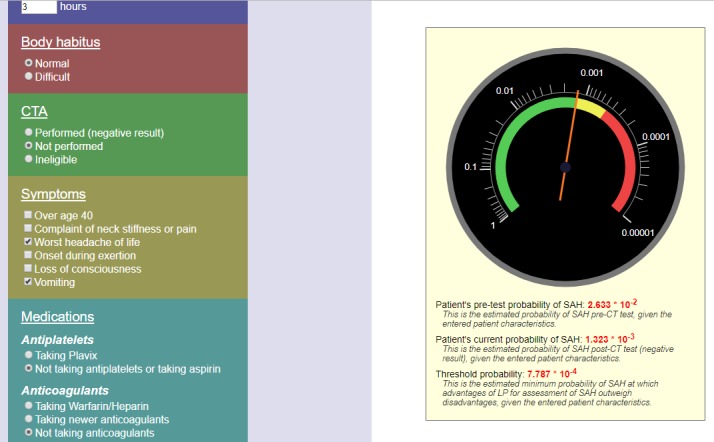
This describes the output of the decision tool for Case 2.

A 26-year-old female with a history of migraine presents with sudden onset, instant peaking headache that started three hours before presentation. She is sensitive to light and sound, and was nauseous until emergency medical services (EMS) arrival and treatment. Her last menstrual period was 40 days ago and she has irregular periods. Her vital signs are stable and her neurological exam is unremarkable. Figure 2 shows the SHARED prototype outputs based on this case. This patient has one risk factor listed in the SAH Ottawa rule—sudden onset with instant peaking pain. Her exam was unremarkable. Based on one risk factor and arrival to the emergency department in three hours, the probability is not high enough to recommend an LP as displayed on the tool. In the yellow zone, physician judgment and/or patient preference will play a role in deciding whether or not to perform the lumbar puncture. If CT angiogram is negative, the tool does not recommend lumbar puncture (Figure 2).

## Conclusions

Limitations and future directions

Here are some limitations of this technical report. First, although subarachnoid hemorrhage is caused by aneurysmal bleed commonly, peri-mesencephalic bleed also results in subarachnoid hemorrhage in 10% of the patients. While aneurysmal bleeds require endovascular treatments, the peri-mesencephalic bleeds require symptom control only. However, these conditions are often not presented separately in studies and therefore, we did not report risk estimates based on disease-specific outcomes. Second, our tool is based on diagnostic accuracy reported in previous studies. Therefore, any limitations of those studies would get carried over to the estimates used in this tool. Third, this tool is a prototype and future directions include estimation of tool inputs in a national sample, validation in an external cohort and development and design of a user-friendly web implementation tool for clinicians.

These preliminary steps will be followed by integration into Health IT and a formal assessment of the web implementation tool in clinical practice.
